# Health system challenges affecting HIV and tuberculosis integration at primary healthcare clinics in Durban, South Africa

**DOI:** 10.4102/phcfm.v11i1.1831

**Published:** 2019-05-09

**Authors:** Dishiki Kalonji, Ozayr H. Mahomed

**Affiliations:** 1 Discipline of Public Health Medicine, University of KwaZulu-Natal, Durban, South Africa

**Keywords:** HIV, tuberculosis, integration, health system, primary healthcare

## Abstract

**Background:**

Tuberculosis (TB) is the most common presenting illness among people living with human immunodeficiency virus (HIV), with co-infection occurring in up to 60% of cases in South Africa. In line with international guidelines, South Africa has adopted an integrated model at primary healthcare level to provide HIV and TB services by the same healthcare provider at the same visit.

**Aim:**

The aim of the study was to conduct a rapid appraisal of integration of HIV and TB services at primary healthcare level in eThekwini District in 2015.

**Setting:**

The study was conducted in 10 provincial primary healthcare clinics in the eThekwini Metropolitan Health District in KwaZulu-Natal Province.

**Methods:**

An observational, cross-sectional study was conducted. Key informant interviews with operational managers and community health workers were conducted, as well as a review of registers and electronic databases for the period of January to March 2015.

**Results:**

Two clinics complied with the mandated integrated model. Three clinics were partially integrated; while five clinics maintained the stand-alone model. Possible constraints included reorganisation of on-site location of services, drug provision, TB infection control and inadequate capacity building, while potential enablers comprised structural infrastructure, staffing ratios and stakeholder engagement.

**Conclusion:**

HIV and TB integration is suboptimal and will need to be improved by addressing the systemic challenges affecting health service delivery, including strengthening supervision, training and the implementation of a change management programme.

## Introduction

Tuberculosis (TB) is the most common presenting illness among people living with human immunodeficiency virus (PLWHIV), including those who are taking antiretroviral therapy (ART). Globally there were an estimated 1.2 million human immunodeficiency virus (HIV)-positive new TB cases in 2014, with approximately 74% of the burden being in sub-Saharan Africa.^1^ Tuberculosis is the leading cause of death among PLWHIV, accounting for some 390 000 HIV-associated TB deaths in 2014 (a 32% reduction since 2004).^2^

South Africa (SA)^3^ is ranked as the country with the second highest incidence rate of TB per 100 000 population globally^3^ but the sixth highest in terms of absolute numbers. Furthermore, out of the 450 000 incident cases in South Africa in 2012, the World Health Organization (WHO) estimated that about 270 000 (60%) people have both HIV and TB infection, ranking SA as the country with the highest burden of HIV–TB co-infection in absolute numbers but second highest in terms of number of co-infected cases per capita.^4^ In South Africa, TB accounted for the third highest number of deaths in 2014 (5.4%; *n* = 33 375), and in combination TB and HIV contributed to 35.6% of all-cause mortality.^4^

KwaZulu-Natal (KZN) carries the largest burden of HIV and related infections in SA, with the HIV–TB co-infection rate estimated at approximately 70%.^5^ In eThekwini Health District, the burden of disease of HIV and TB contributes to 29.6% of years of life lost.^6^ Human immunodeficiency virus and TB contributed to 52% of the total female deaths and 42% of the total male deaths between 2009 and 2014 in eThekwini District.^7^

The HIV epidemic negatively impacts national TB control.^8^ -Co-infection of HIV and TB is regarded as syndemic. The biological interaction between the HIV and TB epidemics results in an exacerbation of the negative health effects of both diseases.^9^ Human immunodeficiency virus alters the natural history of TB; thus, PLWHIV are 26 times (24–28) more likely to develop active TB disease than those without HIV, amplifying the spread of TB. The TB elimination target set for 2050 could be compromised if this dual burden of TB and HIV diseases is not controlled.^9^ To mitigate the dual burden of TB and HIV, the WHO issued a policy on collaborative TB–HIV activities.^8^ These collaborative activities focus on the reduction of the burden of TB in PLWHIV and initiating early ART by intensified case finding for TB, isoniazid preventive therapy (IPT) and TB infection control, as well as the reduction of the burden of HIV in patients with presumptive and diagnosed TB by routine HIV testing with presumptive or diagnosed TB, early initiation of ART and provision of co-trimoxazole preventive therapy (CPT) to all eligible HIV-associated TB clients.^8^

The National Department of Health (NDoH) adopted the WHO policy on collaborative TB–HIV activities^8^ and included these collaborative activities in the 2012–2016 *National Strategic Plan* (NSP) *on HIV, Sexually Transmitted Infections* (STIs) *and TB* with the specific indicators and targets.^10^ Furthermore, in the same year, the NDoH released a practical guide for TB and HIV service integration at primary healthcare (PHC) facilities.^11^

South Africa selected the integrated model, where TB and HIV services are provided at a single facility, at the same time and location.^11^ This is a potentially efficient model for SA to cope with its high HIV, TB and HIV–TB co-infection prevalence, as well as limited human resources.^11^

The 2012 practical guide outlines the key principles to achieve full integration of HIV–TB services at PHC level, such as ongoing training to healthcare workers (HCWs); task-sharing among HCWs of the same level and task-shifting among different levels of HCWs; integration of HIV and TB registers and stationary to prevent duplication and improve the quality of data; integration of TB–HIV services at community level to increase the demand for integrated services at all levels of care, thus accelerating implementation of HIV–TB integrated services; and TB infection control to minimise the risk of nosocomial spread of TB to immunosuppressed people living with HIV or AIDS (PLWHA).^10^

A joint review of the HIV, TB and prevention of mother-to-child transmission programmes conducted by the NDoH in 2014 in 98 facilities nationwide indicated that the SA NSP targets for HIV–TB collaborative activities were not achieved in the financial year 2013–2014.^12^ This 2014 joint review emphasised that although the functional integration of these services was successful at PHC level, the integration was not uniformly distributed across all areas.^12^

**FIGURE 1:**
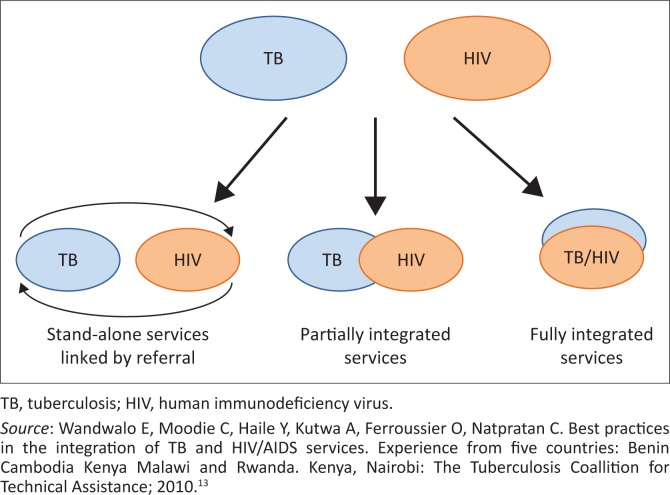
Three models of tuberculosis and human immunodeficiency virus/acquired immune deficiency syndrome service integration.

There are three models for the delivery of integrated HIV and TB services, namely stand-alone (vertical), partially integrated and integrated service models^13^ (Figure 1).

### Stand-alone (vertical) model

Tuberculosis and HIV services are provided at different service delivery points in the same location and linked through a referral system.

*Entry via TB service and referral for HIV testing and care*: TB services refer patients to services providing HIV testing, with or without subsequent HIV care.

*Entry via HIV service and referral for screening, diagnosis and treatment of TB*: HIV services refer PLWHA for TB screening, diagnosis and treatment.

The vertical model is suitable for areas with a low prevalence of both HIV and/or TB diseases.^13^ This model is not the most effective nor efficient model for HIV–TB integrated services, as it requires a strong referral system, it adds additional costs for the patient and it is inconvenient for patients because of the need to visit two clinics. Nevertheless, it serves as a first step in the process of integration.^13^

### Partially integrated model

Some HIV services are provided in clinics and some TB services are provided in HIV clinics, but co-infected patients must still visit two different clinics served by different staff to access full range of HIV and TB services (Figure 1).^13^

*Entry via TB service and referral for HIV care after HIV testing*: Tuberculosis clinics offer HIV testing on-site and refer people found to be HIV-positive for HIV care.

*Entry via HIV service and referral for TB diagnosis and treatment after TB screening*: PLWHA are screened for TB and referred for TB diagnosis and treatment based on the outcome of the screening.

The partially integrated model is the most common model of TB–HIV service integration in most high TB–HIV prevalence countries and settings.^13,14^ This model is appropriate at hospital or health centre level where TB and HIV services are both available but full integration is not possible.^14^ Achievements in Malawi showed an increase in HIV testing of TB patients from 59% to 83%; furthermore, CPT and ART provision to HIV–TB co-infected patients improved from 88% to 100% and 18% to 25%, respectively.^13^ Similarly, in Kenya, there were higher percentages of HIV testing of TB patients and CPT and ART provision than prior to integration.^13^

### Integrated model

Human immunodeficiency virus and TB services are provided by the same trained healthcare provider at the same visit, a ‘one-stop service’: TB clinic provides HIV treatment; HIV clinic provides TB treatment (Figure 1).^13,14^ This model is considered the most efficient and effective way to provide comprehensive TB–HIV services, and it is appropriate for settings with high TB and HIV prevalence.^13,14^ Results in Malawi have shown an increase of 12% in HIV testing of TB patients, from 85% to 97%, as well as an increase of 19% in ART uptake, from 44% to 63%.^13^ Furthermore, there was acceptability of the integrated services among patients. Similarly, in Kenya, integration of HIV–TB services demonstrated improvements in HIV testing of TB patients and provision of CPT and ART.^13^

The purpose of this study was to conduct a rapid appraisal of integration of HIV–TB services in the provincial PHC clinics in eThekwini District in 2015, with a view to determine the extent of the integration of HIV–TB services at facility and community level and describe factors promoting and inhibiting integration of services.

## Research methods and design

### Study design

A retrospective, observational, cross-sectional study design was conducted.

### Study setting

The study was conducted in the eThekwini Metropolitan Health District in KwaZulu-Natal Province. The eThekwini District is densely populated (3 464 205), with the greatest concentrations of the population settling in the South Region (41%). Primary healthcare services are offered at 110 clinics (43 provincial and 57 municipal clinics).

### Study population, sample and size

The target population comprised all provincial PHC clinics in eThekwini Health District. Ten facilities (three best, three intermediate and four poor performing facilities) were purposively sampled by the PHC supervisors to accommodate each subdistrict as well as to contain facilities that achieved or exceeded the TB performance indicators, in addition to facilities that achieved some of the targets and others that did not achieve any of the targets in 2013–2014.^15^

### Data collection

Primary data on HIV and TB services at both facility and community levels were obtained through key informant interviews with the operational managers (OMs) and community health worker (CHW) supervisors during clinic site visits. A set of standardised data collection tools were designed by the researcher to ensure an appraisal of the type, extent and service delivery gaps of integrated HIV–TB services at PHC clinics in 2015. The data collection tools were critiqued and assessed for their validity by the NDoH HIV–TB programme and a panel of technical advisors. The first tool was administered to OMs and the second tool to CHWs. The tools consisted of structured questionnaires with predominantly closed questions and a few open-ended questions.

#### Facility-level data

The tool provided information on services offered; facility infrastructure; service organisation; policies and guidelines; clinical records; facility organisation; process of care; staff rotation and scheduling; human resources; equipment; medication and non-medical supplies; laboratory and other supplies; monitoring and reporting; and communication, referrals and social mobilisation.

#### Community health workers

The tool focused on service provision; training received; campaigns conducted; collaboration with non-governmental organisations (NGOs); and challenges experienced.

Secondary data was obtained by inspection of District Health Information System statistics, registers (TB, IPT, pre-ART and ART) and the electronic databases TIER.net and ETR.net. A data extraction form was used to collect data from the registers. This data included statistics on HIV-positive and TB patients for the period of the last quarter of the financial year 2014–2015, from 01 January 2015 to 31 March 2015. Clinical records of patients within the same period of time were analysed.

### Data analysis

Once the data was collected, the hard copies were stored in a locked file cabinet. The data on the hard copies were transferred to a Microsoft Excel spreadsheet with the same codes as the hard copies. Updated versions of the data did not overwrite the older versions but were renamed using codes to ensure easy data tracking. The Excel spreadsheet was only accessible to the research team. Double entry of the data was done by the researcher and the research assistants to ensure correct data entry and to maintain the integrity of the data. Descriptive statistics were computed, using the STATA software system, version 13.0. Descriptive analysis of variables was expressed as frequencies and percentages for categorical variables.

#### Analysis of the open-ended questions

The principal investigator analysed the open-ended questions themes, and emerging themes were identified and coded. The results were reported as a percentage of facilities that responded with a certain thematic code.

### Ethical considerations

Informed consent was obtained from the participants. Ethical approval to conduct this study was obtained from the Biomedical Research Ethics Committee of the University of KwaZulu-Natal (KZN) (reference number BE257/16). Permission to conduct the study was obtained from the eThekwini District Health Office and the KZN Provincial Department of Health research unit.

## Results

### Human immunodeficiency virus–tuberculosis collaborative activities

#### Facility level

Only two clinics complied with the mandated integrated model for HIV–TB services. Three clinics were partially integrated. The remaining five clinics were still implementing a stand-alone model (Table 1).

**TABLE 1:** Frequency table depicting integration model in the selected primary healthcare clinics in eThekwini Health District, Durban, in 2015.

Integration model	Clinics	
	*n*	%
Stand-alone	5	50
Partially integrated	3	30
Fully integrated	2	20

#### Community level

The overall integration of HIV–TB services at community level is suboptimal. In the financial year 2014–2015, seven clinics conducted awareness campaigns; however, these were focused on vertical programmes. All the clinics have established linkages with community traditional healers to promote HIV–TB integration. One clinic (1/9) established community support groups and community adherence clubs focused on ART. Three clinics have support groups focused on vertical programmes. None of the clinics provide CPT and IPT at a community level. Challenges reported by the CHWs included staff shortage, high workload and lack of personal protection equipment against TB infection.

### Factors influencing human immunodeficiency virus–tuberculosis integration

#### Healthcare workforce

**Availability**: The ratio of professional nurses (PNs) per 100 000 outpatient department (OPD) population was consistent across four clinics ranging between 10 and 20; however the ratio of PNs per 100 000 catchment population varied across clinics, with the majority of clinics with ratios above 30 PNs per 100 000 catchment population.

**Training**: Capacity building for HIV–TB integration was inadequate at the selected PHC clinics. Fewer than 50% of PNs were trained in nurse-initiated management of ART (NIMART) at six clinics. Similarly, fewer than 50% of nurses were trained regarding the national TB guidelines. The majority of clinics trained fewer than 25% of HCWs regarding TB infection control (Table 2).

**TABLE 2:** Percentage of trained healthcare workers at selected primary healthcare clinics in eThekwini Health District, Durban, 2015.

Variable†	Clinics									
	≤ 25		26–50		51–75		76–100		Missing data	
N	%	*N*	%	*N*	%	*N*	%	*N*	%
NIMART	1	11	5	56	1	11	2	22	1	10
2014 TB Management	7	78	1	11	0	0	1	11	1	10
IPC 2007	6	86	0	0	0	0	1	14	3	30

HCWs, healthcare workers; NIMART, nurse-initiated management of ART; TB, tuberculosis; IPC, infection prevention and control.

† , Percentage of HCWs trained regarding the following guidelines.

Seventy per cent of clinics rotate staff across different services every 6 months.

Challenges for staff rotation across different services were attributed to deficiency in skills (80%); staff shortage (50%); and high turnover of staff, absenteeism and personal preferences (30%).

### Medicine supply and equipment

Eighty per cent of clinics experienced a stock-out of ART drugs during the review period, while 30% of clinics reported a TB medication stock-out. In relation to TB care, all clinics highlighted stock-outs of sputum containers and N95 masks, compromising TB screening and infection control. Regarding HIV care, stock-outs of HIV testing kits and ART impede early HIV testing and early initiation of ART.

### Patient records and information system

All clinics are equipped with registers pertaining to TB, TB identification, and HIV counselling and testing. Ninety per cent of the clinics are equipped with pre-ART and IPT registers. However, ART registers are available in 80% of clinics. Only 20% of the clinics are using one fully integrated file per patient. TIER.net was operational at nine clinics; however, 40% of clinics were not using ETR.net as yet.

### Governance and leadership

Only three clinics had a copy of the NSP. The majority of clinics briefed the clinic staff, support partners and clinic committee on HIV–TB integration (see Table 3).

**TABLE 3:** Frequency table relating activities implemented to engage relevant stakeholders at selected primary healthcare clinics in eThekwini Health District, Durban, 2015.

Activities	Clinics	
	*N*	%
**Briefing of staff on HIV/TB integration**	10	100
**HIV–TB integration team**	9	90
**Briefing of clinic committee on HIV–TB integration**	8	80
**Briefing of NGOs and CBOs on HIV–TB integration**	10	100
**HIV–TB integration meetings**		
Staff (monthly)	10	100
With NGOs, CBO, HBC (monthly)	5	50

HIV–TB, human immunodeficiency virus–tuberculosis; NGOs, non-governmental organisations; CBOs, community-based organisations; HBC, home-based carers.

### Tuberculosis infection control

The TB infection control practices were well implemented in the clinics, with over 80% of clinics complying with each activity. Only one activity, triage, had fewer than 90% clinics compliant (see Table 4).

**TABLE 4:** Frequency table demonstrating the tuberculosis infection control practices at selected primary healthcare clinics in eThekwini Health District, Durban, 2015.

IPC activity	Clinics	
	*N*	%
Triage	8	80
Cough hygiene	9	90
Respiratory hygiene	10	100
Ventilated sputum collection area	10	100
Open windows	10	100
Fans	9	90

IPC, infection prevention and control.

#### Infrastructure

The infrastructure in 50% of clinics in the current study comprised seven or more consultation rooms, with three clinics having ten or more rooms. Furthermore, the number of consultation rooms in the two integrated clinics was 6 and 10. Infrastructure challenges were noted to be barriers. Twenty per cent of clinics attributed the lack of integration of files to infrastructural challenges.

## Discussion

South Africa adopted the full integrated model for HIV–TB services. In the current study, only 20% of clinics had established a fully integrated service model, while 50% clinics were still implementing a stand-alone model. The successful integration of HIV–TB services is dependent on infrastructure, staff numbers and capacity, TB infection control, drug provision and stakeholder engagement.^13^

### Infrastructure

Previous studies indicate that three to four consultation rooms are required for integrated HIV–TB care.^13^ Although the clinics had four or more consultation rooms, 90% of the clinics considered the number of consultation rooms insufficient to provide HIV–TB integrated services. Infrastructure appeared to be a key constraint for integration; a similar finding in the 2014 joint review discussed infrastructural challenges compromising effective TB infection control.^12^ However, a possible explanation points to difficulties reorganising the location of the services to enhance integration as the historical mechanism of providing TB–HIV services as vertical services located in different places at facility level is an obstacle for the provision of integrated TB–HIV services.^16^ Infrastructural challenges may inhibit the implementation of a fully integrated model for HIV–TB services and maintain the current challenges of loss to follow-up, long queues, poor adherence to treatment by patients and patients’ dissatisfaction with the current fragmented service delivery.^16^

### Human resources

#### Availability of staff

The staffing ratio of PNs per 100 000 OPD population were similar among the clinics. In contrast, a CHW shortage was present at all clinics, compromising adequate coverage of households. Therefore, staff numbers were only assessed as a constraint for integration at community level. This constraint was also highlighted in a 2012 South African study that interviewed 29 health managers and NGOs, which revealed human resource capacity as a major barrier at community level, reflecting the increased volume of HIV-associated TB cases, funding restraints hindering the creation of posts and difficulties recruiting and retaining staff.^16^ Furthermore, the clinics experienced difficulties rotating staff regularly because of staff preferences, work teams and shifts, skills shortage and absenteeism. The long periods between staff rotation do not allow staff to practice the skills gained from in-service or formal training, thus hindering task-sharing and efficient integration of HIV–TB services.

**Capacity building**: Capacity building for integrated HIV–TB care was inadequate for NIMART, TB infection and TB management, potentially preventing task-shifting from doctors to nurses as mandated by national guidelines^10,16^ and down-referral to community level because of lack of standardised training. These constraints of limited task-shifting and -sharing are mentioned in other South African studies, such as the 2015 qualitative study on clinicians’ perceptions and patients’ experiences of ART integration in PHC clinics, which stated that training was inadequate for PHC skills, national ART guidelines and NIMART, and a 2012 qualitative study on health system barriers to implementation of collaborative TB and HIV activities that referred to inadequate NIMART training.^16,17^

Capacity building and availability of staff are likely constraints for integration at both facility and community levels. The low number of trained healthcare providers compounded by the high volume of patients experienced by most clinics could lead to poor quality of care, high turnover of staff and burnout, and significant staff absenteeism, hence counteracting the potential benefits of fully integrated HIV–TB care. These findings are echoed in the 2014 joint review^12^ and a qualitative study conducted in Sisonke District in KwaZulu-Natal.^16^

### Tuberculosis infection control

The fear of TB infection spread could be a possible constraint. Forty per cent of clinics stated that triaging was conducted but not consistently, a constraint in keeping with a 2013 South African study across 127 PHC clinics in three districts, which found that 48.8% (*N* = 127 PHC clinics) did not separate coughing patients from other patients.^18^ All the clinics indicated stock-outs of N95 masks, a challenge consistent with findings from the 2014 joint review^12^ and the previous highlighted 2013 South African study in three districts.^18^ Moreover, the lack of N95 masks affected collaborative activities at community level. The risk of TB spread is further exacerbated with the inadequate IPC training of HCWs, consistent with a 2013 South African study that revealed that only 33% PNs and 7.2% of CHWs had received training on TB infection control.^18^ Poor IPC will compromise national efforts to control the TB epidemic. Furthermore, PLWHIV are at an increased risk of TB infection acquisition and mortality compared to the general population. Therefore, fully integrated HIV–TB services necessitate effective TB infection control to prevent nosocomial transmission of TB as highlighted in the Extensively drug-resistant TB (XDR-TB) outbreak in Tugela Ferry in KwaZulu-Natal in 2006.^19^

### Medicine supply

Medicine supply challenges included stock-outs of ART and TB treatment as indicated by 80% and 30% of clinics, respectively, potentially inhibiting integration of HIV–TB management as highlighted in a 2011 South African case study on obstacles and solutions regarding HIV–TB integration.^19^

### Governance and leadership

All the clinics engaged their staff, clinic committee and partners in the process of integration of HIV–TB services. In spite of this level of stakeholder engagement, half of the clinics did not complete the process of integration. Stakeholder engagement at community level was mainly focused on vertical programmes, a constraint discussed in the 2014 joint review, where many facilities missed opportunities to integrate TB and HIV community-based activities.^12^ In addition, there was inadequate establishment of community support groups, in contrast to the 2014 Joint Review, which stated that there was good practice of support and capacity building to local community organisations through NGOs in some of the provinces and facilities visited.^12^ The lack of advocacy, communication and social mobilisation for integrated HIV–TB services at community level will inhibit creating a demand for integrated HIV–TB services at all levels of care, thus impeding the integration process.^6^

### Implications of study findings

A systematic review conducted on observational studies using secondary data has found several benefits from integration of TB–HIV integration in sub-Saharan Africa.^20^ Implementing integration at facility and community level requires a combination of *clinical integration* (TB and HIV care, treatment, diagnostic testing and health education activities taking place concurrently) and *organisational integration* (facility-level resources [e.g. staff, infrastructure, space, patient files and data systems] and processes [e.g. patient flow] are integrated).^21^ As this study has highlighted, full integration is being hampered by organisational factors. However, these factors are not insurmountable, as many of the highlighted challenges require minor modification in processes at facility level. Although the South African guidelines explain what is needed to integrate TB and HIV services, the ‘how’ aspect is lacking. A quality improvement model using facility-based multidisciplinary teams together with change management at facility and community level is required to ensure implementation and sustainability of HIV–TB integration.

#### Study limitations

Although due diligence was exercised in maintaining the scientific integrity of this appraisal, it is limited by the quality of data, the selection bias because of non-probability sampling and most importantly the small sample size. These limitations compromise the generalisability of these findings to other settings and contexts.

The quality of data was a limitation to the study because of separate registers and notes for ART and TB care requiring the same information. Frequent data recording results in incomplete data recording in registers. In addition, although TIER.net (for ART clients) is implemented at all clinics, ETR.net (for registered TB clients) is still not implemented at 40% of clinics.

## Conclusion and recommendations

Four factors were identified as possible constraints for integration of HIV–TB services, namely reorganisation of location of services, medicine supply, TB infection control and inadequate capacity building. The factors identified as possible promoters included structural infrastructure, staffing ratios and stakeholder engagement.

Human immunodeficiency virus and TB integration will need to be improved by addressing the systemic challenges affecting health service delivery, including strengthening supervision, training, and medicine supply and adopting a quality improvement model.
